# Residual Stress Relief in High-Strength Steel Welded Joints: Creep-Based Material Modeling and Post-Weld Treatment Simulation

**DOI:** 10.3390/ma19091696

**Published:** 2026-04-23

**Authors:** Penglong Ding, Silu Zheng, Jiahe Zhou, Xiatao Tang, Huina Shan, Chuanyang Lu, Wenjian Zheng, Xuhui Gong, Jiajia Niu, Lianyong Xu

**Affiliations:** 1 School of Materials Science and Engineering, Tianjin University, Tianjin 300350, China; dingpenglong@126.com (P.D.); xulianyong@tju.edu.cn (L.X.); 2 Luoyang Ship Material Research Institute, Luoyang 471023, China; gongxuhui725@sina.com (X.G.); niujia9876@163.com (J.N.); 3 Institute of Process Equipment and Control Engineering, College of Mechanical Engineering, Zhejiang University of Technology, Hangzhou 310014, China; zhoujiahe@zjut.edu.cn (J.Z.); zwj0322@zjut.edu.cn (W.Z.); 4 Zhejiang Academy of Special Equipment Science, Hangzhou 310020, China; txt@zjut.edu.cn (X.T.); zjtjshn@163.com (H.S.); 5 National Key Laboratory of Marine Corrosion and Protection, Luoyang 471000, China

**Keywords:** high strength steel welded joint, finite element simulation, residual stress, creep behavior, post-weld treatment

## Abstract

Residual stress is an inherent consequence of the welding process and can significantly compromise the structural integrity of welded components. To clarify the high-temperature creep damage evolution of the 600 MPa-grade ship hull structural steel base metal, high-temperature creep tests were conducted, aiming to improve the understanding of its deformation behavior and to support reliable numerical predictions. The experimentally calibrated creep constitutive model was subsequently integrated into finite element simulations to analyze the residual stress evolution in welded joints and to quantitatively evaluate the effects of post-weld heat treatment (PWHT) and hammer peening. The results indicted that, within 450–550 °C, creep deformation of the steel was dominated by dislocation glide and climb, while creep damage was mainly associated with void and crack formation. The simulation results revealed that residual stresses were predominantly concentrated in the weld metal and the heat-affected zone, with the peak von Mises stress in the as-welded joint reaching 686.5 MPa, exceeding the material’s yield strength at the simulated temperature. PWHT exhibited superior stress-relief effectiveness compared with hammer peening, markedly reducing the peak residual stress. Moreover, the stress-relief behavior showed a nonlinear dependence on both holding time and heat-treatment temperature. In contrast, hammer peening produced a localized stress-relief effect, confined primarily to the mechanically impacted region. These findings provided a theoretical foundation for optimizing post-weld treatment strategies to mitigate residual stress in the high strength steel welded joints.

## 1. Introduction

The 600 MPa-grade ship hull structural steel is a high-performance alloy widely employed in aerospace, petrochemical, and deep-sea engineering applications, serving as a critical structural material for aircraft and spacecraft components, high-pressure vessels and pipelines, and pressure-resistant hulls of offshore platforms [1,2,3]. In engineering practice, multi-pass groove welding is commonly used on steel components, generating complex thermal cycles and mechanical constraints that induce substantial residual stresses in both the weld metal and heat-affected zone [4,5]. In particular, X-groove butt welding with alternating multi-pass deposition is widely adopted in thick-plate structures owing to its effectiveness in mitigating welding distortion while ensuring full penetration. However, this welding configuration also induces pronounced residual stresses, particularly concentrated near the weld toe and root, necessitating effective post-weld stress mitigation [6]. Various post-weld treatment techniques, most notably post-weld heat treatment (PWHT) and mechanical hammering, are commonly employed in engineering practice to mitigate residual stresses [7,8,9,10]. PWHT is a conventional and effective method for residual stress relief, typically performed through annealing or tempering processes. The procedure involves uniformly heating the welded component from ambient temperature to a designated elevated temperature, maintaining that temperature for several hours, and subsequently allowing the component to cool slowly. This controlled thermal cycle promotes stress relaxation via microstructural recovery and creep mechanisms. Although effective, PWHT requires specialized heating equipment, stringent thermal control, and is associated with high energy consumption. In contrast, the hammering technique relies on localized mechanical impacts applied to regions with high residual stress. Repeated striking introduces compressive stresses into the surface layer and induces localized plastic deformation, thereby reducing the peak residual stress and homogenizing the overall stress distribution. This redistribution enhances resistance to brittle fracture and improves the structural reliability of the joint.

Over the past few decades, post-weld techniques for alleviating residual stress in welded joints have attracted considerable research attention [11,12,13,14,15]. Wu et al. [16] investigated the effect of local heat treatment on residual stress in AH36 thick-plate weldments through combined experimental and numerical approaches. Their results showed that local heat treatment effectively reduced residual stress, and that both annealing temperature and holding time exert decisive control over stress elimination. Kumar et al. [17] conducted welding experiments and finite element (FE) simulations on Alloy 617/10Cr steel and found that circumferential and transverse residual stresses peaked along the joint centerline. They further reported that PWHT at 680 °C for 24 h significantly reduced the residual stress level. Hu et al. [18] incorporated the Norton–Bailey model and a creep model incorporating back stress into the simulation of the heat treatment process. Their results indicated that the model yields more accurate predictions of stress relaxation and achieves a greater reduction in residual stresses, particularly at lower temperatures. Wang et al. [19] simulated the welding and PWHT processes of Ti62A alloy plates, revealing the influence mechanisms of heat-treatment parameters on residual stress evolution. They found that both the heating rate and the heat-treatment temperature exerted pronounced effects on residual stress relief. In addition, Yadav et al. [20] compared the effects of varying heat-treatment temperatures on the microstructure and properties of aluminum alloy 2024, demonstrating that temperature-dependent precipitate evolution markedly influenced the performance of welded joints.

Post-weld hammer peening and related mechanical treatments have also been shown to be effective in relieving residual stresses. Existing studies indicate that hammering introduces beneficial compressive stresses into the weld surface, thereby reducing tensile residual stresses and improving the overall stress state of welded components [21]. Zhou et al. [22] simulated residual stress reduction in cross-welded structures using alternating inter-layer hammering and full inter-layer hammer peening. Their results demonstrated that both approaches decreased the residual stresses, with inter-layer hammer peening achieving a more pronounced stress-relief effect. Zhu et al. [23] combined experiments and simulations to investigate the evolution of welding residual stress in multi-layer, multi-pass A350-LF2 steel joints. They reported that when welding occurred within the phase-transformation temperature range, the resulting microstructural changes contributed to a reduction in residual stress. Dai et al. [24] conducted FE simulations of post-weld hammering on SM490 steel and found that the overall reduction in residual stress was primarily attributable to compressive stresses induced by the hammer peening process. Although numerous studies have investigated the effects of PWHT and hammer peening on residual stress mitigation, several limitations remain. Most existing numerical simulations focus on a single stress-relief method, and systematic comparative analyses of PWHT and hammer peening within identical welding configurations remain scarce. Moreover, despite the fact that the creep behavior of the base material plays a crucial role in stress relaxation during high-temperature treatment, it is frequently simplified or omitted in existing simulation models. The combined effect of creep deformation and different post-weld treatment methods on residual-stress redistribution in the 600 MPa-grade ship hull structural steel has not been comprehensively clarified.

To address these gaps, this study investigated the residual-stress reduction in the 600 MPa-grade ship hull structural welded joints subjected to either PWHT or hammer peening. High-temperature creep tests were conducted on the base metal to characterize its creep deformation and damage mechanisms, and the resulting creep parameters were incorporated into a finite element framework. Subsequently, coupled thermo-mechanical simulations of welding, PWHT, and hammer peening were performed to evaluate and compare their stress-relief effectiveness and underlying mechanisms. The findings provided theoretical guidance and technical support for optimizing post-weld stress-relief strategies in engineering applications of 600 MPa-grade ship hull structural steels.

## 2. Materials and Methods

### 2.1. Creep Tests

#### 2.1.1. The 600 MPa-Grade Ship Hull Structural Steel

**Figure 1:**
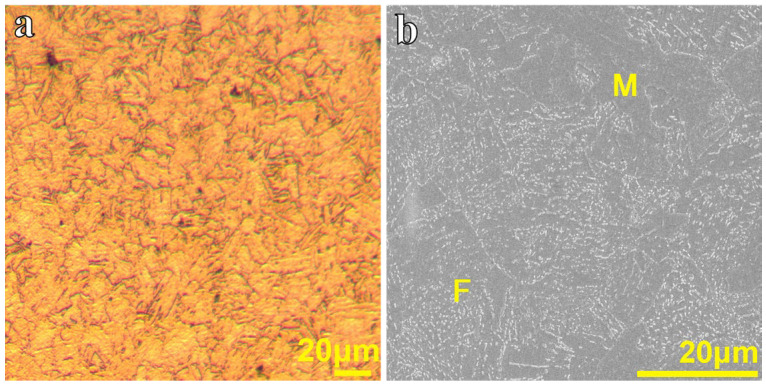
Microstructures of the steel: (**a**) OM image and (**b**) SEM image.

The microstructure of the material is shown in Figure 1, where the matrix consists predominantly of martensite (*M*) and ferrite (*F*). The main chemical composition of the steel and its mechanical properties at different temperatures are listed in Table 1 and Table 2, respectively.

#### 2.1.2. Testing Process

**Figure 2:**
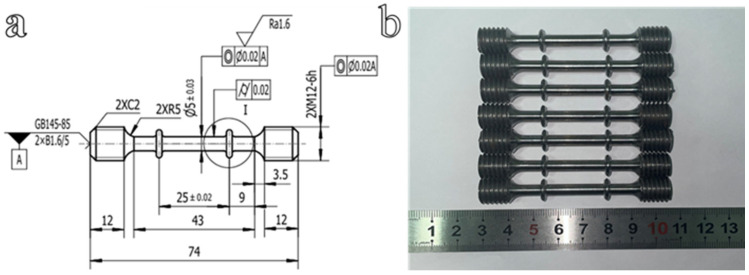
(**a**) Dimensions of the creep test specimen (mm). (**b**) Photograph of the creep test specimen.

To accurately simulate the PWHT process and its effect on residual stress relaxation, it is necessary to characterize the high-temperature creep behavior of the base material. The dimensions of the creep specimens and the physical appearance of the samples are shown in Figure 2. Standard round-bar specimens with a gauge length of 25 mm and a diameter of 5 mm were employed for high-temperature creep tests. In Figure 2a, A is the common axis determined by the center holes at both ends of the specimen. Tests were conducted using an RDL-100 electronic creep testing machine (China Machine Test Equipment Co., Ltd., Changchun, Jilin Province, China). Thermocouples were attached to the upper, middle, and lower sections of each specimen to monitor the temperature distribution. Specimens were heated at a rate of 10 K/min to the target temperature, held isothermally for 1 h to ensure thermal equilibrium, and then subjected to creep loading. Each test condition was repeated three times to ensure reproducibility. Considering the temperature range relevant to the subsequent PWHT simulations, the creep tests were conducted at 450 °C, 500 °C, and 550 °C. For each temperature level, the applied creep stresses were set to 1.0, 0.8, 0.6, and 0.4 times the corresponding yield strength (*R*_p0.2_). The specific test parameters are listed in Table 3.

The raw material and test specimens were sectioned along the axial direction by wire electrical discharge machining to obtain slices ~2 mm in thickness for microstructural examination. The samples were mechanically ground and subsequently polished using a 0.5 μm diamond suspension. After cleaning and drying, the specimens were electrolytically etched using a 4 vol.% nitric acid solution in ethanol. The microstructures were then examined using an optical microscope (OM, Aosvi M230-3M50; Shenzhen Longma Visual Light Source Co., Ltd., Shenzhen, Guangdong Province, China) and a scanning electron microscope (SEM, FEI Quanta 200F; FEI Company, Hillsboro, OR, USA).

### 2.2. Finite Element Analysis

#### 2.2.1. Finite Element Model

**Figure 3:**
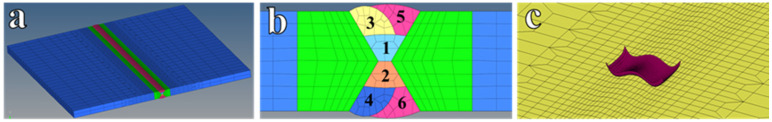
(**a**) Three-dimensional (3D) finite element model and welding mesh. (**b**) Cross-sectional finite element mesh of the weld. (**c**) Hammer peening model for post-weld treatment. Different colors represent different mesh partitions, and numbers 1–6 indicate the weld pass sequence.

The commercial finite element software MSC Marc (Marc 2016) was employed to simulate the evolution and subsequent mitigation of welding residual stresses in steel butt-welded joints. A three-dimensional (3D) model was developed to simulate the sequential multi-pass welding process, as shown in Figure 3a,b. The model comprised two identical plates (300 mm × 150 mm × 12 mm), joined via an X-shaped groove configuration. Cross welding was adopted because it can effectively mitigate welding deformation, and the subsequent pass thermally reheats the prior pass, thereby promoting localized stress relief and reducing peak tensile stresses near the first-welded side. Accordingly, a symmetric and alternating welding sequence was applied on both sides of the joint, with two layers and three passes deposited on each side of the bevel. The FE model of the weld cross-section is illustrated in Figure 3b. Different colors represent different mesh partitions, and numbers 1–6 indicate the weld pass sequence. The welded joint was discretized using a structured hexahedral mesh. Finer elements were assigned to the weld and heat-affected zone, while coarser elements were used in regions farther away from the weld. Smooth mesh transition was implemented between fine and coarse regions. The final model comprised 27,249 nodes and 27,100 elements. The hammer peening model used for post-weld mechanical treatment is shown in Figure 3c.

#### 2.2.2. Thermal Properties and Heat-Source Model

During welding, the temperature undergoes rapid and complex variations due to localized heat input and transient conduction. Given that the filler metal and base metal are both 600 MPa-grade ship hull structural steel, exhibiting identical chemical compositions and mechanical properties. Therefore, the same material properties were assigned to both the filler metal and the base metal in the simulation. The thermal properties of the 600 MPa-grade ship hull structural steel used in the numerical analysis are listed in Table 4.

**Figure 4:**
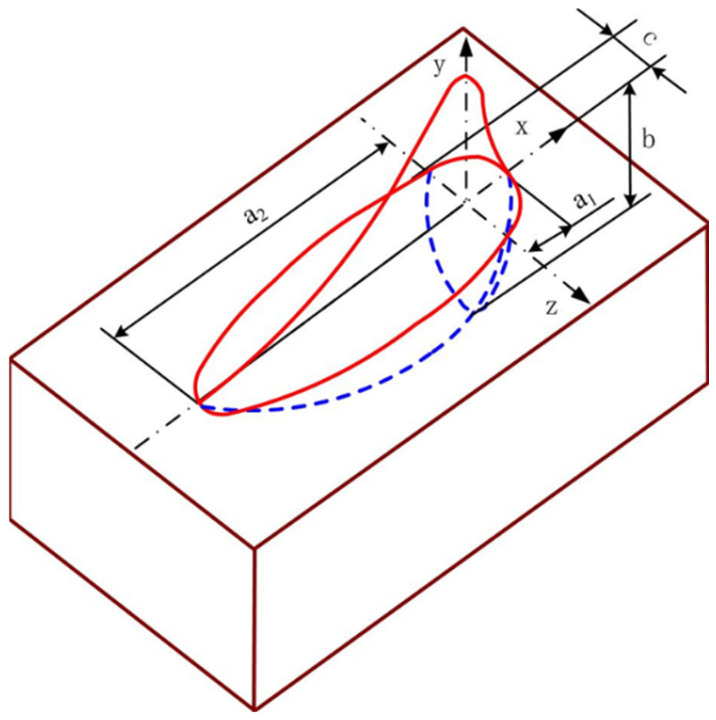
Heat-source distribution diagram of the Goldak double-ellipsoid heat-source model. The blue line and the red line represent the front contour and the rear contour of the double ellipsoid model, respectively.

The welding heat-source model constitutes a fundamental component of thermo-mechanical welding simulations, directly governing the accuracy of predicted temperature fields, phase transformations, and residual stress distributions. For groove welds, the heat source was implemented as an internal heat generation term within the weld elements. Heat input was applied in the form of a volumetric heat generation rate, and the element birth-and-death technique was employed to simulate the progressive deposition of weld metal. To accurately reproduce the weld penetration characteristics, the Goldak double-ellipsoidal heat-source model was employed [25]. The normalized heat-flux distribution of the Goldak model is illustrated in Figure 4. As shown, the molten pool is divided into two regions (the front and rear ellipsoids) to represent the asymmetric distribution of heat during arc welding. The blue line and the red line represent the front contour and the rear contour of the double ellipsoid model, respectively. The mathematical formulation of the double-ellipsoidal heat source is provided in the following Equations.

The heat-source distribution equation for the front half of the ellipsoid:

(1) $$q\left(x,y,z,t\right)=\frac{6\sqrt{3}f_{f}Q}{a_{1}bc\pi \sqrt{\pi }}e^{-3x^{2}/{a_{1}}^{2}}e^{-3y^{2}/b^{2}}e^{-3z^{2}/c^{2}}$$

The heat-source distribution equation for the back half of the ellipsoid:

(2) $$q\left(x,y,z,t\right)=\frac{6\sqrt{3}f_{r}Q}{a_{2}bc\pi \sqrt{\pi }}e^{-3x^{2}/{a_{2}}^{2}}e^{-3y^{2}/b^{2}}e^{-3z^{2}/c^{2}}$$

(3) Q=ηUI

In Equations (1) and (2), the *f*_f_ and *f*_r_ represent the distribution indexes of the total input power in the front and back parts of the melting pool, respectively, and *f*_f_ + *f*_r_ = 2. *a*_1_, *a*_2_, *b* and *c* are the shape parameters of the welding heat source (unit: mm), which can be obtained from the welding pass distribution mechanism. *x*, *y*, *z* are the space position coordinates (mm) of the heat source at time *t* (s). *Q* (W) is the effective heat input. *η* is the welding thermal efficiency. *U* (V) is the welding voltage, and *I* (A) is the welding current.

#### 2.2.3. Boundary Condition

The model was initialized at a uniform ambient temperature of 20 °C for the welding simulation. Prior to welding, the groove region and a 150 mm wide zone extending symmetrically on both sides were uniformly preheated to ~200 °C, and the inter-pass temperature was maintained at the same level throughout the welding process. Convective heat transfer was applied to represent the welding thermal boundary conditions. During welding, heat dissipation occurs primarily through radiation and convection between the workpiece surface and the surrounding environment, with radiation contributing to the dominant portion of heat loss. To ensure computational efficiency without compromising physical fidelity, the radiation and convection effects were combined into an equivalent overall heat transfer coefficient in the simulation [26]. The surface heat loss rate (*Q*_s_) can be expressed using Equations (4) and (5):

(4) $$Q_{s}=\beta \left(T-T_{0}\right)$$

(5) $$\beta =\beta_{e}+\beta_{c}$$

where *T* (°C) and *T*_0_ (°C) represent the surface temperature of the welded part and the temperature of the surrounding medium, respectively. *β* is the surface heat transfer coefficient. *β*_e_ is the convective heat transfer coefficient. *β*_c_ represents the radiative heat transfer coefficient. The units of the three coefficients are W/m^2^/°C. In the simulation, fixed boundary constraints were applied to both sides of the model along the longitudinal direction. During the heat treatment process, the rigid displacement of the structure was restrained, and the heating and cooling rates did not exceed 100 °C/h. The peak temperatures were held at 450 °C, 500 °C, and 550 °C, respectively, with holding times of 4–6 h. For the post-weld hammer peening simulation, arc-shaped hammer heads were applied to impact the weld toe regions on both outer surfaces of the joint. The curved surface of the hammer head was defined as a rigid body, whereas the welded plate was defined as a deformable body. Contact interaction was established between the rigid and deformable bodies, and the friction coefficient was set to 0.1.

## 3. Results and Discussion

### 3.1. Analysis of Creep Test Results

#### 3.1.1. Creep Test Results

**Figure 5:**
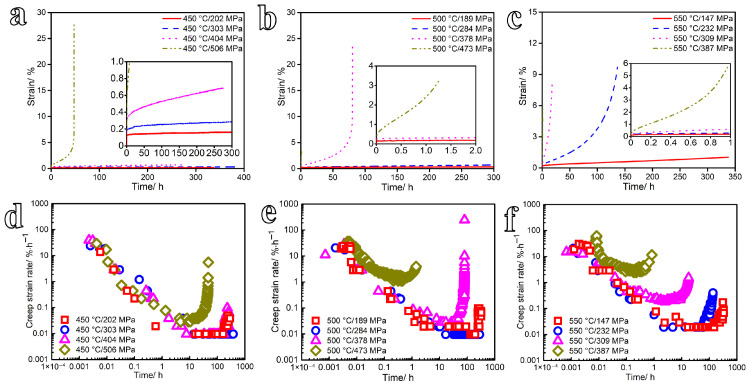
Typical (**a**–**c**) strain-time curves (The insets in Figure 5 show the magnified results of local areas.) and (**d**–**f**) creep strain rate as a function of time for the 600 MPa-grade ship hull structural steel tested at 450–550 °C under stresses ranging from 147 to 506 MPa.

The typical strain-time curves and the relationships between creep strain rate and time for the tests conducted at 450–550 °C under stresses ranging from 147 to 506 MPa are shown in Figure 5. The creep strain, creep time, and steady-state creep rate under different test conditions are summarized in Table 5. As shown in Figure 5a–c, the creep response exhibits the classical three-stage behavior: the primary (decelerating) stage, the secondary (steady-state) stage, and the tertiary (accelerating) stage. Correspondingly, the creep strain rate declines rapidly during primary creep, attains a near-constant minimum value during secondary creep, and surges exponentially in the tertiary regime prior to fracture, as illustrated in Figure 5d–f. The results further show that the steady-state creep rate and the accumulated creep strain both increase with increasing applied stress at a given temperature and test duration. Under the same stress ratio (the ratio of applied stress to yield strength) and test duration, the steady-state creep rate and creep strain also increase with rising temperature. Notably, at all tested temperatures, the steady-state creep stage under stresses equal to *R*_p0.2_ is significantly shortened compared with the other conditions. This acceleration arises because the steady-state creep rate at the *R*_p0.2_ stress level is relatively high, causing rapid deformation of the specimen and thereby reducing the duration of the steady-state stage.

#### 3.1.2. Microstructural Examination

**Figure 6:**
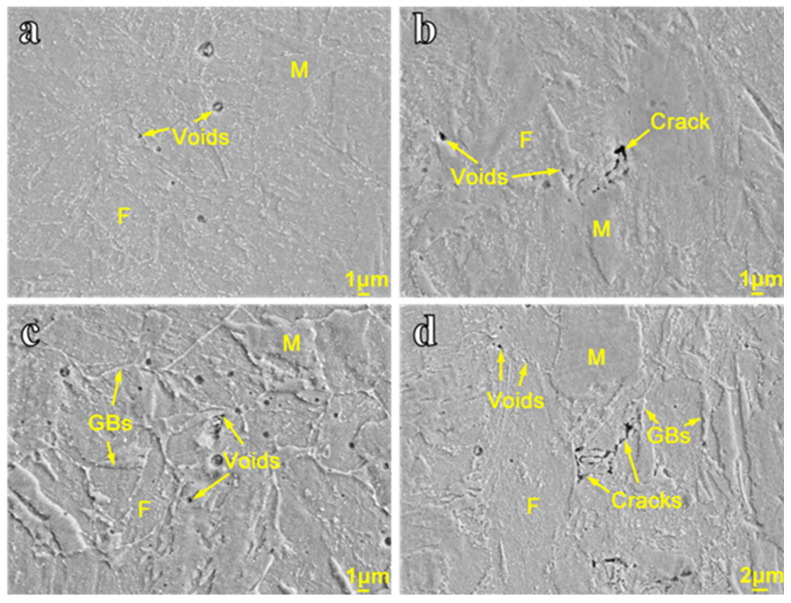
SEM images of the specimens tested at (**a**) 450 °C/404 MPa, (**b**) 500 °C/378 MPa, (**c**) 550 °C/147 MPa, and (**d**) 550 °C/309 MPa.

Microstructure critically governs both the dominant deformation mechanisms and the prevailing damage initiation and propagation pathways during high-temperature creep. Figure 6 presents the SEM images of the specimens tested at 450 °C/404 MPa, 500 °C/378 MPa, 550 °C/147 MPa, and 550 °C/309 MPa. The results show that the microstructure after creep does not exhibit significant changes compared with the original material. The microstructure remains dominated by martensite and ferrite, along with a considerable amount of white carbide precipitates. Creep voids and cracks are observed under all temperature and stress conditions [27]. At 550 °C/147 MPa and 550 °C/309 MPa, void nucleation occurs preferentially at grain boundaries (GBs). These observations indicate that creep voids nucleate, grow, and coalesce at GBs, eventually forming microcracks. As creep exposure continues, the microcracks propagate along GBs and develop into microcracks, leading to intergranular fracture.

#### 3.1.3. Creep Model

Creep deformation is governed by temperature, stress, and time. During PWHT, creep strain contributes to the relaxation of welding residual stresses; therefore, the high-temperature creep effect must be incorporated into the numerical simulation. To capture this response, a creep constitutive model was incorporated into the finite element framework. Fundamentally, the creep strain (*ε*) can be expressed as a function of time (*t*), temperature (*T*), and applied stress (*σ*):

(6) $$\varepsilon =f_{1}\left(t\right)f_{2}\left(T\right)f_{3}\left(\sigma \right)$$

The creep acceleration stage leads to rapid plastic deformation, whereas the overall creep behavior is predominantly governed by the steady-state stage. Therefore, the Norton model is adopted to describe creep behavior in the simulation of post-weld heat treatment. The simplified form of the model can be expressed as follows [28]:

(7) $$\dot{\varepsilon }=A\left(T\right)\sigma^{n}$$

(8) $$A\left(T\right)=A_{0}exp\left(-\frac{Q_{s}}{RT}\right)$$

where $\dot{\varepsilon }$ is the steady-state creep rate (%/h). σ is the stress (MPa). n is the stress index. *A* and *A*_0_ are material constants. *R* is the molar gas constant (8.314 J/mol/K). *Q*_s_ is the creep activation energy (J/mol). *T* is the test temperature (°C).

**Figure 7:**
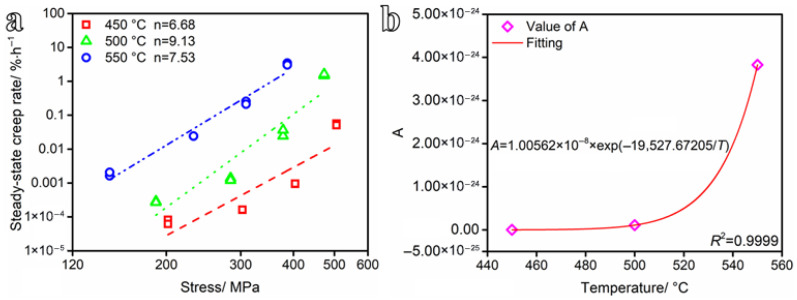
(**a**) Dependence of steady-state creep rate on applied stress, and (**b**) variation in the Norton parameter *A* with temperature.

According to the test results, the logarithm of Equation (7) was applied, and the linear fitting results of the relation between the stress (ln*σ*) and the steady-state creep rate ($\ln\dot{\varepsilon }$) are shown in Figure 7a. The parameters of *A*, *n* and *Q*_s_ at different temperatures can be obtained from Figure 7a. The creep deformation mechanism can be characterized by the n. The deformation mechanism of metal materials is mainly diffusion creep (*n* = 1), dislocation glide (*n* = 2), and dislocation climb (*n* = 3–8). When *n* ≥ 10, the creep is dominated by both dislocation glide and climb [29]. Figure 7a shows that at temperatures of 450 °C, 500 °C, and 550 °C, the stress exponent *n* is 6.68, 9.13, and 7.53, respectively. Accordingly, the dominant creep deformation mechanisms of the steel at these temperatures are identified as follows: dislocation climb at 450 °C, dislocation glide and climb at 500 °C, and dislocation climb at 550 °C. The parameter *A* of the Norton model generally varies with temperature. To establish the relationship between *A* and temperature, the average value of n at different temperatures is taken to obtain the corresponding parameter *A*, and then the relationship with temperature is fitted. The expression of material constant *A* and temperature can be described by nonlinear fitting, and the coefficient of determination (*R*^2^) is 0.9999, as shown in Figure 7b. The creep model expression is shown in Equation (9).

(9) $$\dot{\varepsilon }=1.00562\times 10^{-8}exp(-\frac{19527.67205}{T})\sigma^{7.78}$$

The assumptions and limitations associated with the adopted creep model are clarified as follows: (1) the Norton creep law assumes that secondary (steady-state) creep dominates the deformation behavior, while primary (transient) and tertiary (accelerating) creep stages are neglected. This simplification is reasonable for PWHT, as the process is conducted at a constant elevated temperature over an extended duration, during which steady-state creep is expected to prevail; (2) the temperature dependence of the material parameter *A*(T) is empirically fitted based on experimental data obtained for the specific steel grade within the temperature range of 450–550 °C. Therefore, extrapolation beyond this range remains unvalidated and should be approached with caution unless supported by additional experimental evidence; (3) the model assumes a constant microstructure throughout the creep process, thereby neglecting potential microstructural evolution. This assumption is generally acceptable for typical PWHT durations, where significant microstructural changes are limited; (4) isotropic creep behavior is assumed, and the welded joint is treated as a macroscopically homogeneous material; and (5) the model evaluates creep strain without incorporating any damage evolution parameters. This simplification is appropriate for simulations focused on residual stress relaxation, where damage accumulation is not the primary concern. Within these assumptions and limitations, the Norton model provides a convenient and computationally efficient framework for predicting stress relaxation behavior during PWHT.

### 3.2. Analysis of Finite Element Simulation Results

#### 3.2.1. Residual Stress Distribution in the Welded Joint

**Figure 8:**
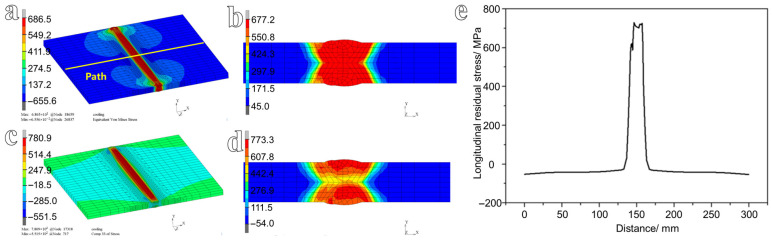
Contour plots of the simulated residual stress: von Mises stress distribution of (**a**) the welded joint and (**b**) the cross-section, longitudinal residual stress distribution in (**c**) the welded joint and (**d**) the cross-section, and (**e**) longitudinal residual stress distribution along the specified path.

The calibrated creep model was implemented via a user-defined material subroutine (UMAT) and integrated into the subsequent heat-treatment simulation to capture the relaxation of welding residual stress induced by creep. To systematically compare the efficacy of PWHT and mechanical hammer peening in mitigating residual stresses within steel welded joints, a comprehensive thermo-mechanical welding process simulation was first performed to establish the baseline as-welded residual-stress distribution. The von Mises stress is a key parameter for evaluating material strength and structural integrity under complex loading conditions, and it was used to identify regions of stress concentration and potential failure locations [30]. In the welding process, the longitudinal residual stress *S*_11_, which is perpendicular to the welding direction, is of primary concern in this study. The residual-stress distribution obtained from the welding simulation is shown in Figure 8. The results indicate that the residual stress is highly non-uniform, exhibiting a pronounced stress gradient across the welded joint. High residual stresses are mainly concentrated in the weld metal and adjacent regions, extending into the outer areas of the heat-affected zone. The peak von Mises stress reaches 686.5 MPa, exceeding the yield strength (*R*_p0.2_) of the steel. The primary contributing factor is strain hardening. During multi-pass welding, the material in the weld and heat-affected zone (HAZ) undergoes repeated thermal cycles involving rapid heating and cooling, which induce significant plastic deformation. As plastic strain accumulates, the yield strength of the material increases due to strain hardening [31,32,33], thereby elevating the stress level that the material can sustain. In addition, the imposed boundary conditions play a critical role. Fixed constraints applied along the longitudinal direction restrict the free thermal shrinkage of the structure during cooling. As a result, the shrinkage tendency is suppressed, and substantial elastic strains are generated in the weld and HAZ to accommodate this constraint. The combined effects of strain hardening and constrained deformation ultimately lead to the development of high residual stresses. In contrast, the regions farther from the weld exhibit relatively low residual stresses, as shown in Figure 8a. Due to the displacement constraints imposed at both ends of the weld, the gradient of von Mises stress is steep near the constrained boundaries, whereas the gradient in the mid-region of the weld is comparatively mild. The von Mises stress distribution on the cross-section is shown in Figure 8b, revealing a maximum value of 677.2 MPa localized at the weld toe region. The longitudinal stress distribution contour plots of the welded structure and the cross-section are presented in Figure 8c and Figure 8d, respectively, with maximum stresses of 780.9 MPa and 773.3 MPa, respectively. To compare the variations in residual stress between the untreated and heat-treated conditions, the maximum longitudinal stress obtained from the cross-section of the welded structure was selected. For the untreated condition, the simulated peak longitudinal residual stress was 773.3 MPa. The minimum stresses are −55.2 MPa and −54.0 MPa, respectively. That indicates that both tensile and compressive stresses exist in the welded structure. The fundamental reason for that is the mutual opposition between the “thermal expansion and contraction” behavior due to local heating and cooling and the overall constraint of the structure, ultimately leading to static equilibrium. The longitudinal stress distribution along the outer-surface path (indicated in Figure 8a) is plotted in Figure 8e. The results demonstrate that the longitudinal residual stress reaches its maximum at the weld location, while the stress level in the base metal regions is markedly lower, quantitatively consistent with the contour plots of the residual-stress field.

#### 3.2.2. Influence of Heat Treatment Process on Residual Stress

**Figure 9:**
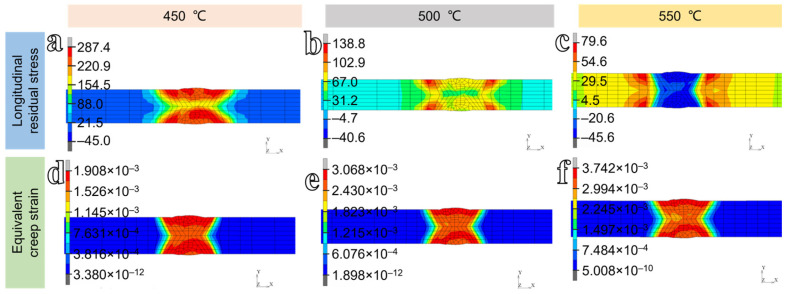
(**a**–**c**) Longitudinal residual stress distributions, and (**d**–**f**) equivalent creep strain distributions after heat treatment at different temperatures.

To elucidate the influence of PWHT temperature on the welding residual stress, the longitudinal stress distribution on the cross-section after heat treatment is analyzed. The cross-sectional longitudinal stress contour maps and equivalent creep strain contour maps of the welded structure under different heat treatment temperatures are shown in Figure 9. The results reveal that the overall distribution patterns of longitudinal stress after heat treatment at various temperatures remain similar, with high residual stresses persisting at the weld centerline and adjacent heat-affected zone. In addition, the equivalent creep strain increases progressively with increasing heat-treatment temperature.

Compared with the untreated condition, the peak longitudinal residual stresses after heat treatment at 450 °C, 500 °C, and 550 °C are 287.4 MPa, 138.8 MPa, and 79.6 MPa, respectively. The corresponding reduction ratios are 62.83%, 82.05%, and 89.71%. Similarly, multi-pass welded joints of S690QL1 high-strength steel were subjected to PWHT at holding temperatures of 400 °C, 530 °C, 550 °C, and 600 °C. Quantitative measurements revealed that the residual stress in the base-metal region decreased by 72%, 81%, 93%, and 100% relative to the as-welded condition, respectively [34]. These results demonstrate a strong monotonic correlation between PWHT temperature and residual stress mitigation, indicating that stress relaxation becomes progressively more complete with increasing thermal activation energy. The difference between the maximum and minimum residual stresses decreases from 827.3 MPa in the as-welded condition to 332.4 MPa, 179.4 MPa, and 125.2 MPa following PWHT at 450 °C, 500 °C, and 550 °C, corresponding to reductions of 59.82%, 78.32%, and 84.87%, respectively. Following PWHT, compressive residual stresses developed in all cases. The magnitudes of the compressive stresses formed at 450 °C, 500 °C, and 550 °C are 45 MPa, 41 MPa, and 46 MPa, respectively. The associated creep deformations at these temperatures are 0.19%, 0.30%, and 0.37%.

**Figure 10:**
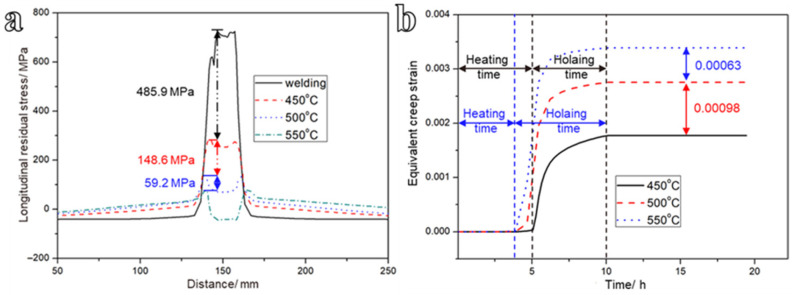
(**a**) Comparison of longitudinal residual stress along the specified path under different heat treatment temperatures, and (**b**) corresponding creep strain results.

The longitudinal stress results along the specified path at the three heat treatment temperatures are shown in Figure 10a. The residual stress attenuation decreases from 148.6 MPa at 450 °C to 59.2 MPa at 550 °C, indicating progressively diminishing marginal returns in stress relief with increasing PWHT temperature. Notably, even at 450 °C, the reduction in welding residual stress is already substantial. The variation in creep strain along the same path during heat treatment is presented in Figure 10b, indicating that negligible equivalent creep strain accumulates below the material-specific creep activation threshold temperature. Upon reaching the target PWHT temperature, the viscoplastic deformation initiates, leading to measurable accumulation of equivalent creep strain. At each PWHT temperature, the strain asymptotically approaches its saturation value within 5–6 h of holding, demonstrating that residual stress relaxation has reached a quasi-steady state. The incremental equivalent creep strain also decreases from 0.00098 to 0.00063 as the PWHT temperature rises from 450 °C to 500 °C, reflecting a reduction in the instantaneous creep rate at elevated temperatures due to progressive stress relaxation. These results demonstrate that the stress-relief effect achieved by heat treatment is significant and becomes more pronounced at higher temperatures. Furthermore, the results validate that adopting Norton creep law in FE simulation to model creep during PWHT can provide valid stress results [35].

The above results indicated that both the reduction in residual stress and the accumulation of creep strain increase with rising heat treatment temperature. This indicates that high-temperature heat treatment markedly lowers the yield strength of the material and simultaneously activates atomic diffusion and dislocation motion. Consequently, the high residual stresses inherited from the welded joint are effectively released, thereby promoting creep plastic deformation, resulting in substantial stress relaxation. Moreover, the influence of heat treatment temperature on residual-stress reduction is greater than that of holding time. To achieve an efficient and sufficient release of residual stress, the PWHT temperature for the 600 MPa-grade ship hull structural steel should be set at 550 °C, with a holding time of 6 h.

#### 3.2.3. Influence of Post-Welding Hammer Peening on Residual Stress

**Figure 11:**
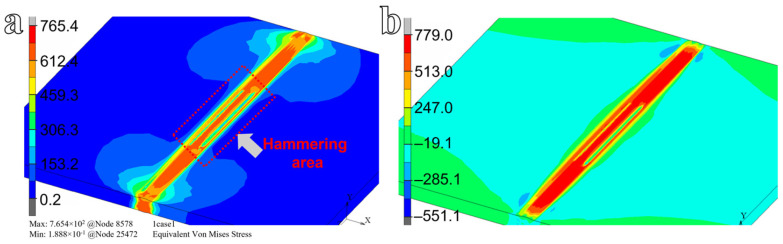
(**a**) Von Mises stress distribution, and (**b**) longitudinal residual stress distribution after post-weld hammering.

**Figure 12:**
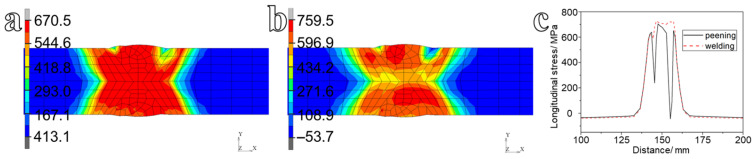
(**a**) Von Mises stress distribution and (**b**) longitudinal residual stress distribution on the cross-section, and (**c**) variation in residual stress along the specified path before and after hammering.

Figure 11 presents the residual stress distribution following post-weld hammer peening. Relative to the as-welded condition, the von Mises stress and longitudinal residual stress after hammering increase to 765.4 MPa and 779 MPa, corresponding to a rise of 11.49% and a reduction of 0.25%, respectively. Although global extremum values exhibit limited change, the contour plots (Figure 11a,b) reveal a pronounced localized reduction in both stress components within the hammered weld zone, most notably at the weld toe, where compressive peening-induced stresses effectively counteract tensile welding residuals, thereby alleviating local stress concentration and enhancing stress uniformity [36]. The stress distribution across the joint cross-section is shown in Figure 12. The maximum von Mises stress and longitudinal residual stress on the cross-section after hammering are 670.5 MPa and 759.5 MPa respectively, which decrease by 0.99% and 1.78% respectively, as shown in Figure 12a,b. To compare the effect of residual stress reduction, the stress distribution along the typical path before and after hammering is shown in Figure 12c. A pronounced stress-relief effect is observed at the hammering location. However, the affected region is relatively small and remains confined to the impacted zone. In the surrounding regions, changes in residual stress are minimal. This behavior is attributed to the compressive stress introduced by hammering, which counteracts the tensile welding residual stress and thereby reduces the local stress level. Nonetheless, due to the limited area of hammer impact, the extent of the stress-relief zone is restricted.

In summary, during the welding of metal structures, the combined effects of mechanical constraints and concentrated heat input lead to the generation of high residual stresses in the weld region. PWHT and hammer peening are both effective methods for reducing these stresses. PWHT eliminates residual stress over a larger area and with better effect than post-weld hammering. Furthermore, the heat treatment temperature has a stronger influence on residual-stress elimination than the holding time. When the heat treatment temperature is 550 °C and the holding time is 6 h, the longitudinal residual stress after welding can be reduced by 89.71%. In practical engineering applications, the selection of residual-stress mitigation methods should consider the combined effects of welding parameters and economic efficiency in order to achieve optimal stress-relief performance at minimal cost.

## 4. Conclusions

In this study, high-temperature creep tests were conducted on the base metal to characterize the creep deformation behavior of the 600 MPa-grade ship hull structural steel. The experimentally calibrated creep constitutive model was subsequently implemented into a Marc user subroutine, on the basis of which a finite element model of the welded joint was established. Simulations of welding, post-weld heat treatment, and hammer peening were then performed to evaluate their effectiveness in relieving residual stresses. The main conclusions were summarized as follows:

(1) Within the temperature range of 450–550 °C, the dominant creep deformation mechanisms of the 600 MPa-grade ship hull structural steel were dislocation glide and climb, while creep damage was primarily characterized by the nucleation and growth of voids and cracks.
(2) The welded joint exhibited a pronounced residual stress gradient. High residual stresses were concentrated in the weld metal and the adjacent heat-affected zone, whereas significantly lower stresses were observed in the remaining regions of the joint.
(3) Post-weld heat treatment demonstrated a strong capability for stress relief. The degree of residual stress reduction increased with the heat-treatment temperature. When the peak temperature exceeded 450 °C, the peak von Mises stress in the welded joint was reduced by more than 62.83%. In contrast, the stress-relief effect of post-weld hammer peening was mainly localized in the treated area, with limited influence on regions beyond the hammered zone.

Finally, it should be noted that the present study focuses on a limited range of PWHT temperatures (450–550 °C). The creep constitutive model was calibrated under constant uniaxial stress (202–506 MPa), and its applicability to complex multi-axial stress states or other materials requires further validation. Future work will extend the model to a wider range of temperatures and stress levels, and also investigate the influence of multi-axial stress state on residual stress relaxation.

## Figures and Tables

**Table 1:** Chemical composition of the steel (wt.%).

Ni	Cr	C	Si	Mn	Mo	P	V	S	Fe
2.80	1.08	0.14	0.37	0.41	0.29	0.02	0.07	0.01	Bal.

**Table 2:** Mechanical properties of the steel at different temperatures.

Temperature/[°C]	100	200	300	400	500	600	700
Young’s modulus/[GPa]	203.4	200.8	194.8	185.5	173.0	157.0	138.6
Yield strength (*R*_P0.2_)/[MPa]	615	576	586	538	473	300	109
Poisson’s ratio	0.3						

**Table 3:** High-temperature creep parameters.

Temperature/[°C]	Stress/[MPa]			
	*R*_p0.2_	0.8 *R*_p0.2_	0.6 *R*_p0.2_	0.4 *R*_p0.2_
450	506	404	303	202
500	473	378	284	189
550	387	309	232	147

**Table 4:** Thermal properties of the 600 MPa-grade ship hull structural steel.

Temperature /[°C]	Coefficient of Thermal Expansion/[°C]	Conductivity /[W/m/°C]	Density /[kg/m^3^]	Specific Heat /[J/kg/°C]	Convective Heat Transfer Coefficient /[W/m^2^/°C]
−100	1.03 × 10^−5^	/	7870	440	15
0	1.03 × 10^−5^	/	7870	440	15
20	1.03 × 10^−5^	35.3	7807	440	15
100	1.36 × 10^−5^	36.4	7870	470	15
200	1.53 × 10^−5^	38.1	7870	520	15
300	1.77 × 10^−5^	39.0	7870	870	15
400	1.62 × 10^−5^	38.5	7870	620	15
500	1.56 × 10^−5^	35.7	7870	660	15
600	1.21 × 10^−5^	34.2	7870	780	15
700	1.17 × 10^−5^	26.7	7870	860	15
800	1.78 × 10^−5^	25.0	7870	800	15
900	2.04 × 10^−5^	25.0	7870	730	15
1000	2.16 × 10^−5^	25.0	7870	730	15
1500	2.46 × 10^−5^	26.8	7870	730	15

**Table 5:** Creep test results of the steel under different conditions.

Temperature /[°C]	Stress /[MPa]	Strain /[%]	Time /[h]	Steady-State Creep Rate /[%/h]
450	202	0.16 ± 0.020	337 ± 0	(7.1948 ± 1.0336) × 10^−5^
450	303	0.29 ± 0.005	404 ± 118	(1.6239 ± 0.0050) × 10^−4^
450	404	0.71 ± 0.060	404 ± 118	(9.2832 ± 0.1198) × 10^−4^
450	506	27.00 ± 1.000	47 ± 2	0.0536 ± 0.0030
500	189	0.31 ± 0.020	337 ± 0	(2.7884 ± 0.0924) × 10^−4^
500	284	0.77 ± 0.015	403 ± 119	0.0013 ± 0.0001
500	378	6.11 ± 0.030	91 ± 10	0.0305 ± 0.0059
500	473	3.20 ± 0.020	2 ± 1	1.5459 ± 0.0689
550	147	0.90 ± 0.100	337 ± 0	0.0018 ± 0.0002
550	232	11.65 ± 1.950	142 ± 5	0.0226 ± 0.0015
550	309	8.63 ± 0.545	17 ± 1	0.2325 ± 0.0220
550	387	5.20 ± 0.635	1 ± 0	3.2328 ± 0.2098

## Data Availability

The original contributions presented in this study are included in the article. Further inquiries can be directed to the corresponding authors.
